# Analysis of the evolution of placental oxidative stress research from a bibliometric perspective

**DOI:** 10.3389/fphar.2024.1475244

**Published:** 2024-10-17

**Authors:** Ailing Chen, Mengyuan Tian, Zouqing Luo, Xiaohui Cao, Yanfang Gu

**Affiliations:** Department of Obstetrics and Gynecology, Women’s Hospital of Jiangnan University, Wuxi Maternity and Child Health Care Hospital, Wuxi, China

**Keywords:** placenta, oxidative stress, bibliometrics, citespace, VOSviewer, emerging topics, research focus

## Abstract

**Background:**

Research on placental oxidative stress is pivotal for comprehending pregnancy-related physiological changes and disease mechanisms. Despite recent advancements, a comprehensive review of current status, hotspots, and trends remains challenging. This bibliometric study systematically analyzes the evolution of placental oxidative stress research, offering a reference for future studies.

**Objective:**

To conduct a comprehensive bibliometric analysis of the literature on placental oxidative stress to identify research hotspots, trends, and key contributors, thereby providing guidance for future research.

**Methods:**

Relevant data were retrieved from the Web of Science Core Collection database and analyzed using VOSviewer, CiteSpace, and the bibliometrix package. An in-depth analysis of 4,796 publications was conducted, focusing on publication year, country/region, institution, author, journal, references, and keywords. Data collection concluded on 29 April 2024.

**Results:**

A total of 4,796 papers were retrieved from 1,173 journals, authored by 18,835 researchers from 4,257 institutions across 103 countries/regions. From 1991 to 2023, annual publications on placental oxidative stress increased from 7 to 359. The United States (1,222 publications, 64,158 citations), the University of Cambridge (125 publications, 13,562 citations), and Graham J. Burton (73 publications, 11,182 citations) were the most productive country, institution, and author, respectively. The journal Placenta had the highest number of publications (329) and citations (17,152), followed by the International Journal of Molecular Sciences (122 publications). The most frequent keywords were “oxidative stress,” “expression,” “pregnancy,” “preeclampsia,” and “lipid peroxidation.” Emerging high-frequency keywords included “gestational diabetes mellitus,” “health,” “autophagy,” “pathophysiology,” “infection,” “preterm birth,” “stem cell,” and “inflammation.”

**Conclusion:**

Over the past 3 decades, research has concentrated on oxidative stress processes, antioxidant mechanisms, pregnancy-related diseases, and gene expression regulation. Current research frontiers involve exploring pathophysiology and mechanisms, assessing emerging risk factors and environmental impacts, advancing cell biology and stem cell research, and understanding the complex interactions of inflammation and immune regulation. These studies elucidate the mechanisms of placental oxidative stress, offering essential scientific evidence for future intervention strategies, therapeutic approaches, and public health policies.

## 1 Introduction

Placental oxidative stress has been a focal point in the forefront of biological and medical research. As the key organ facilitating the exchange of substances between the mother and the fetus, the placenta plays a crucial role in maintaining fetal development and maternal health during pregnancy. Research on the placenta encompasses various aspects, including its structure, function, mechanisms of development, and potential complications (Burton and Jauniaux, 2023; Phengpol et al., 2023; Zhang et al., 2023a; Hu et al., 2020). Among these, the study of oxidative stress within the placenta has drawn significant attention (Phengpol et al., 2023; Zhang et al., 2023a; Hu et al., 2020; Myatt and Cui, 2004). Oxidative stress, characterized by an imbalance between reactive oxygen species (ROS) and antioxidant defenses, is a condition resulting from the disruption of the cellular and extracellular environment (Phengpol et al., 2023; Zhang et al., 2023a; Hu et al., 2020; Burton and Jauniaux, 2011). This condition is closely associated with numerous pregnancy-related disorders, such as preeclampsia (Phengpol et al., 2023; Zhang et al., 2023a; Hu et al., 2020; Roberts and Cooper, 2001; Steegers et al., 2010), gestational diabetes (Phengpol et al., 2023; Zhang et al., 2023a; Hu et al., 2020; Fisher et al., 2021), and intrauterine growth restriction (IUGR) (Phengpol et al., 2023; Zhang et al., 2023a; Hu et al., 2020; Burton and Jauniaux, 2018). Understanding the relationship between oxidative stress and placental function is essential for developing therapeutic strategies to mitigate these adverse outcomes.

Over the past decades, the field of oxidative stress research has made advancements. These progressions are reflected not only in the accumulation of scientific knowledge but also in the continual development of methodological approaches and technological innovations. However, given the vast amount of scientific literature, systematically organizing and analyzing the progress, hotspots, and trends in this field has become a pressing challenge. In this context, bibliometrics offers a novel perspective by employing mathematical and statistical methods to quantitatively analyze scientific literature.

Bibliometric analysis, which involves the quantitative evaluation of published literature, provides valuable insights into the development and trends of a specific research area. By analyzing publication patterns, citation networks, and keyword frequencies, researchers can identify key contributors, emerging themes, and potential gaps in the literature. The objective of this bibliometric study is to perform a comprehensive analysis of the literature on “placental oxidative stress,” uncovering its historical evolution and forecasting future research directions.

We specifically utilized bibliometric methods in conjunction with tools such as the bibliometrix package, CiteSpace, and VOSviewer to analyze publications on “placental oxidative stress” from the Web of Science Core Collection. Our analysis encompasses the distribution of annual publications, countries, institutions, authors, source journals, keyword co-occurrence, and co-citations. The goal of this bibliometric analysis is to gain an in-depth understanding of the current state, hotspots, and future development trends in placental oxidative stress research. This study not only enhances our comprehension of the historical and contemporary landscape of placental oxidative stress research but also provides valuable resources and insights for researchers aiming to navigate and contribute to this dynamic field. Ultimately, our findings aim to guide clinical practice and scientific research in this area.

## 2 Methods

### 2.1 Data collection and retrieval strategy

**FIGURE 1:**
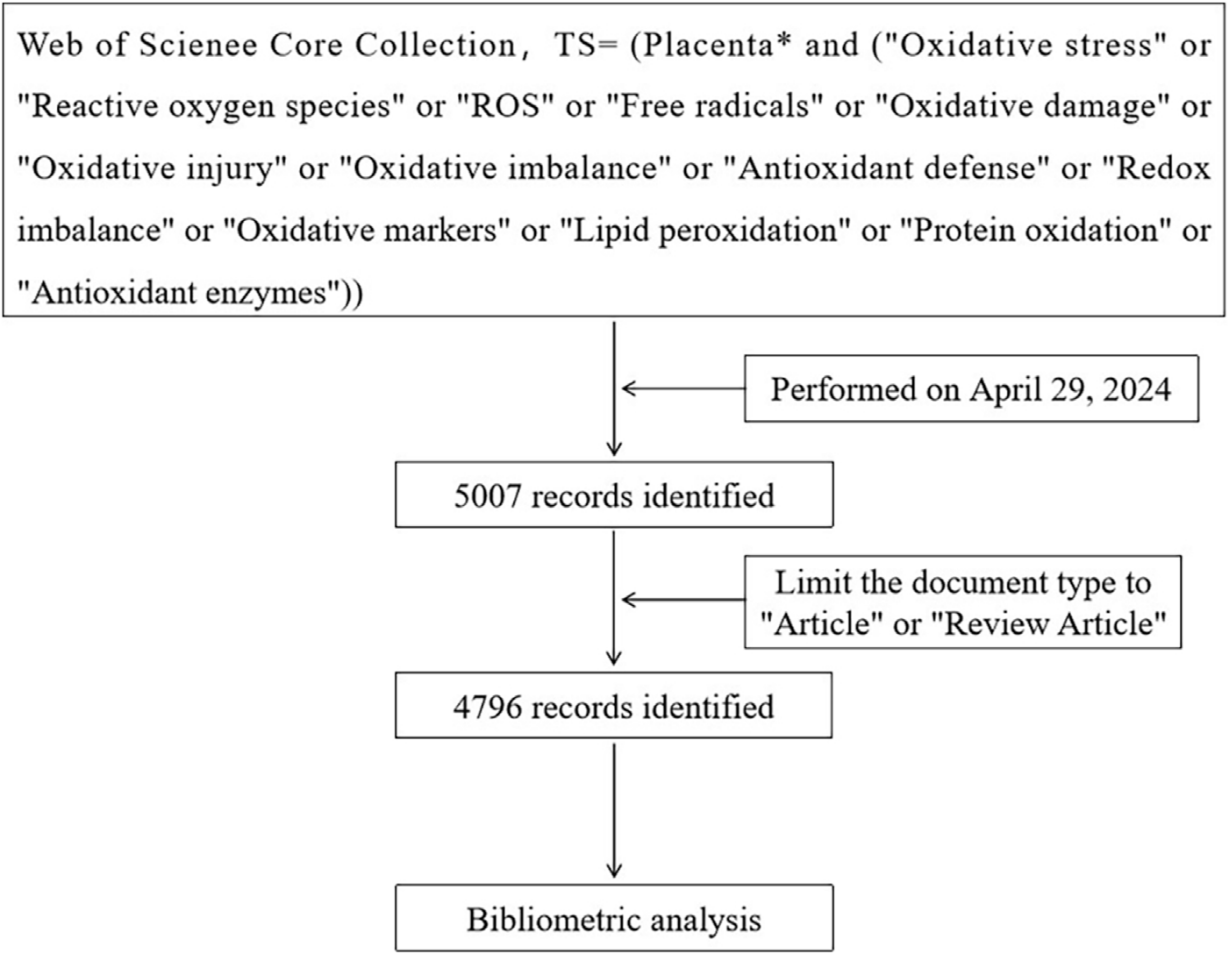
Flow-chart of the search process.

To enhance the representativeness and accessibility of the data, we conducted a literature search in the Web of Science Core Collection on 29 April 2024. Figure 1 illustrates the data collection and retrieval strategy. We specified the search terms using the “Topic” (TS) field, which encompasses the title, abstract, author keywords, and Keywords Plus. The search query was structured as follows: TS = (Placenta* AND (“Oxidative stress” OR “Reactive oxygen species” OR “ROS” OR “Free radicals” OR “Oxidative damage” OR “Oxidative injury” OR “Oxidative imbalance” OR “Antioxidant defense” OR “Redox imbalance” OR “Oxidative markers” OR “Lipid peroxidation” OR “Protein oxidation” OR “Antioxidant enzymes”)). We limited the publication type to articles and reviews, without imposing any time or language restrictions. A total of 4,796 records were retrieved, encompassing publications, authors, countries, institutions, journals, keywords, and citations. These records were exported in the format of complete records.

### 2.2 Data analysis

Bibliometric data analysis was conducted using VOSviewer (v1.6.19), CiteSpace (v6.1. R6 Basic), and the bibliometrix package (version 4.1.3) within the R statistical environment (version 4.3.1). Preliminary descriptive statistics on the number of publications and citations per year, country, and author were generated using the bibliometrix package. Additionally, this package was employed to analyze the distribution of publications, collaboration patterns between countries/regions, authors’ productivity over time, the top 10 highly cited references and co-cited references, trend topic analysis, and word cloud visualization. VOSviewer was utilized for data extraction and visualization of countries, institutions, authors, and keywords. CiteSpace was used to analyze country collaborations, perform cluster analysis of co-cited references, create a dual-map overlay of journals related to “placental oxidative stress,” analyze keyword timeline cluster maps, and detect keyword bursts for the top 25 keywords exhibiting the highest burst strength.

## 3 Results

### 3.1 Annual global publication outputs on placental oxidative stress

**FIGURE 2:**
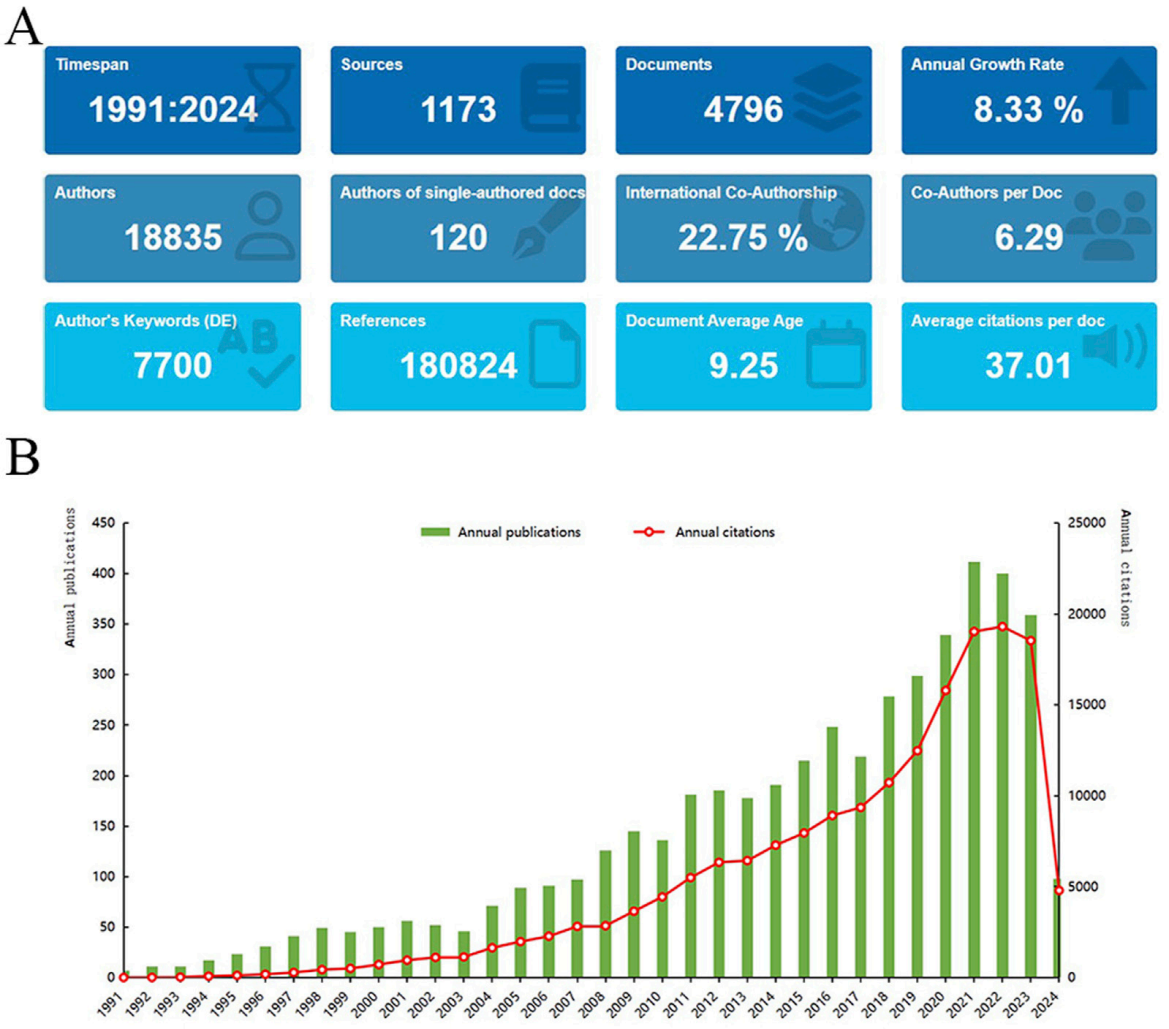
A bibliometric analysis of research on placental oxidative stress. **(A)** Bibliometric Data Overview. **(B)** Distribution of “Placental Oxidative Stress” publications over time.

The earliest publication on “placental oxidative stress” dates back to 1991, with a total of 4,796 articles identified (Figure 2A). Annual publication trends reveal an 8.33% growth rate, indicating a steady increase in interest since 1991 (Figure 2B). Publications increased from 7 to 23 between 1991 and 1995, reflecting early academic interest despite low activity. Between 1996 and 2000, publications increased from 31 to 50, reflecting a surge in research interest. From 2001 to 2010, publication growth accelerated, especially after 2008, marking a phase of high-speed development. During 2011 to 2020, publication numbers remained high with fluctuating increases. In 2021, the number of publications peaked at 412, demonstrating high research activity and academic interest. Despite a slight decline from 2022 to 2023, the count remained substantial, indicating sustained interest. The field has been cited 177,497 times, averaging 37.01 citations per article. The stable upward trend in citations over the past 30 years underscores the growing research interest in “placental oxidative stress.” These findings underscore the field’s significance and provide a foundation for future research directions and strategies.

### 3.2 Distribution and co-authorship of countries/regions

**FIGURE 3:**
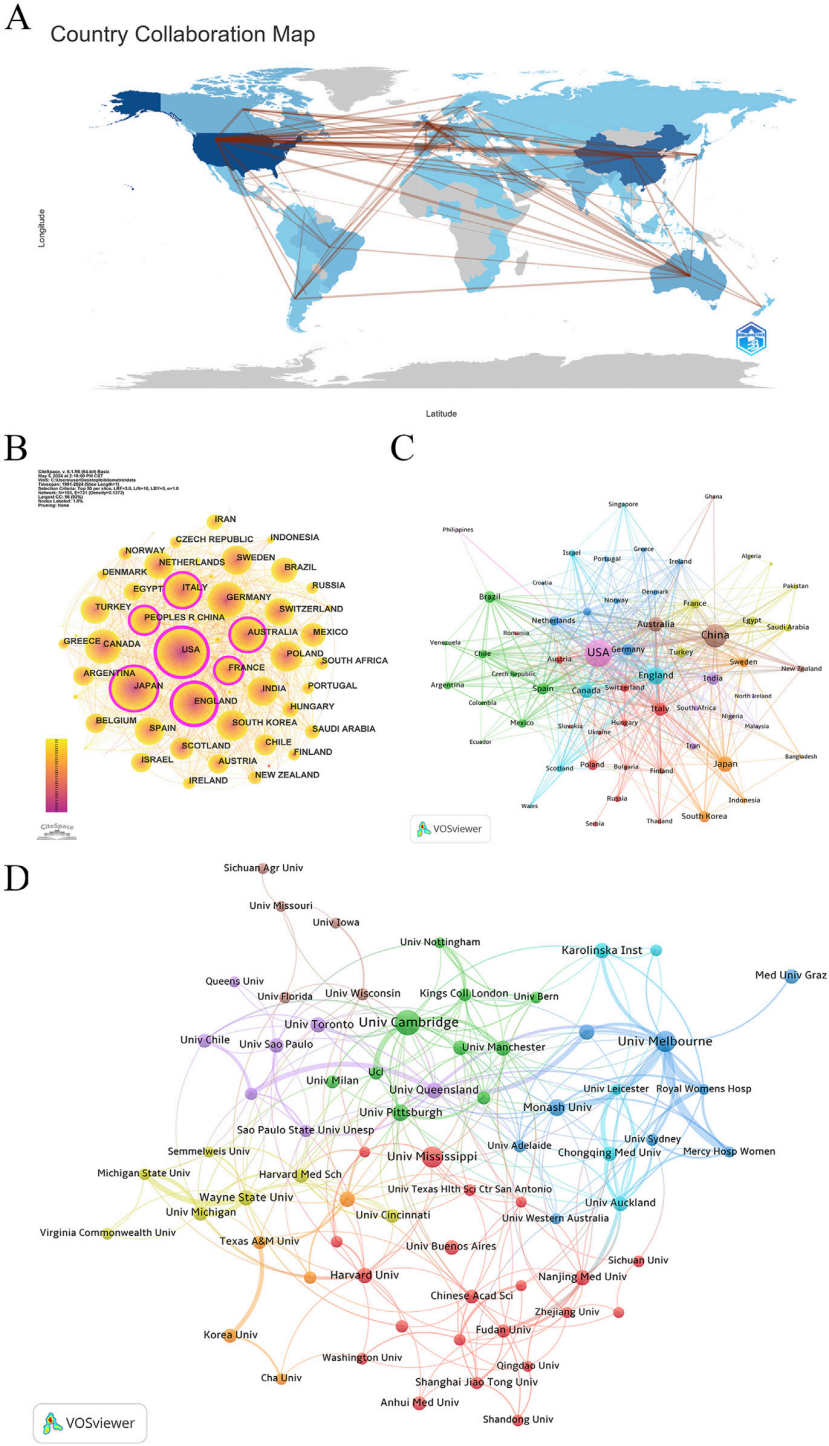
Collaboration network of countries/regions/institutions. **(A)** Distribution and collaboration of publications among countries/regions. **(B)** CiteSpace: visualizing clusters of cooperation among countries/regions. **(C)** VOSviewer: visualizing clusters of cooperation among countries/regions. **(D)** Visualization map of institutional collaboration.

Research on “placental oxidative stress” has emerged as a global hotspot, involving researchers from 103 countries/regions across six continents (Figure 3A). The United States, China, and England lead in publication counts, collectively accounting for over 50% of the total research output, reflecting their significant interest. The H-index rankings also emphasize the United States, England, and Canada as top contributors (Table 1). CiteSpace analysis of international collaboration networks identifies the United States, England, Italy, China, Australia, Japan, and France as central nodes (Figure 3B). VOSviewer analysis of co-authorship, with a minimum of five publications, shows the United States, England, and China dominating in Total Link Strength. England, Canada, and the United States lead in average citations, indicating high research quality and strong international collaboration (Table 1).

**TABLE 1:** Top 10 most productive countries in Placental Oxidative Stress Research.

Rank	Country	Publications n (%)	Total citations	Average citations	H-index	Total link strength	Centrality
1	United States	1222 (25.53)	64,158	52.5025	119	612	0.28
2	China	810 (16.92)	14,718	18.1704	54	249	0.14
3	England	396 (8.27)	32,904	83.0909	88	374	0.23
4	Japan	305 (6.37)	9185	30.1148	50	107	0.12
5	Australia	280 (5.85)	11,833	42.2607	54	242	0.11
6	Canada	231 (4.83)	16,613	71.9177	60	168	0.02
7	Italy	201 (4.2)	7458	37.1045	46	175	0.19
8	India	177 (3.7)	3403	19.226	32	44	0.01
9	Brazil	167 (3.49)	2873	17.2036	29	97	0.03
10	Spain	166 (3.47)	4728	28.4819	33	188	0.08

VOSviewer further classifies countries into nine collaboration clusters based on co-authorship strength (Figure 3C). The United States leads the violet cluster, with strong ties to China, England, Canada, Australia, and the Philippines. China heads the dark brown cluster, collaborating closely with Australia, New Zealand, and Ghana. England’s turquoise cluster includes Canada, Scotland, Israel, Singapore, and Wales. Japan’s orange cluster features collaborations with South Korea, Sweden, Indonesia, and Bangladesh. Italy, Poland, and Switzerland are central to the largest cluster, the dark red cluster, comprising 13 countries. India leads the blue-purple cluster, collaborating with Iran, South Africa, and Nigeria. Brazil forms the green cluster with Spain, Chile, and Mexico. Germany’s deep blue cluster includes the Netherlands, Belgium, and Norway, while Turkey’s dark yellow-green cluster collaborates with France, Egypt, and Saudi Arabia.

These findings underscore the extensive international collaboration and influential contributions in placental oxidative stress research, providing a foundation for future research directions and collaborations.

### 3.3 Distribution of research institutions and authors

#### 3.3.1 Distribution of research institutions

Using VOSviewer, the study analyzed institutional co-authorship in research on placental oxidative stress. The analysis applied “Association Strength” with a minimum document threshold of 20, examining 4,257 institutions, of which 77 met the criteria. Table 2 shows that the University of Cambridge led with 125 papers (2.61%), followed by the University of Melbourne (88 papers, 1.83%), the University of Mississippi (83 papers, 1.73%), Monash University (53 papers, 1.11%), and the University of Pittsburgh (51 papers, 1.06%). Notably, four of the top ten institutions are based in Australia, and three are in the United States. The University of Pittsburgh recorded the highest average citations per paper (140.24), followed by the University of Cambridge (108.50) and Harvard University (91.81). The University of Melbourne exhibited the highest total link strength (107), reflecting its strong collaborative connections within this research field.

**TABLE 2:** Top 10 most productive institutions in the research on Placental Oxidative Stress.

Rank	Institution	Country	Publications n (%)	Total citations	Average citations	Total link strength
1	University of Cambridge	England	125 (2.61)	13,562	108.50	55
2	University of Melbourne	Australia	88 (1.83)	3504	39.82	107
3	University of Mississippi	United States	83 (1.73)	3821	46.04	8
4	Monash University	Australia	53 (1.11)	1417	26.74	38
5	University of Pittsburgh	United States	51 (1.06)	7152	140.24	21
6	University of Queensland	Australia	48 (1)	1956	40.75	54
7	Karolinska Institutet	Sweden	45 (0.94)	2366	52.58	18
8	University of Toronto	Canada	44 (0.92)	2441	55.48	15
9	Griffith University	Australia	42 (0.88)	1350	32.14	28
10	Harvard University	United States	42 (0.88)	3856	91.81	22

Figure 3D categorizes the leading institutions into eight clusters. The green cluster includes the University of Cambridge, the University of Pittsburgh, the University of Manchester, the University of Alberta, and the University of Milan. The dark blue cluster groups Australian institutions such as the University of Melbourne, Monash University, and Griffith University. The dark red cluster features the University of Mississippi, Harvard University, Nanjing Medical University, and the Universidad de Buenos Aires. The blue-purple cluster comprises the University of Queensland, the University of Toronto, the University of São Paulo, and the University of Chile. The blue-green cluster includes Karolinska Institutet, Chongqing Medical University, and the University of Auckland. The dark yellow-green cluster brings together Wayne State University, the University of Michigan, and Harvard Medical School. The orange cluster includes the University of Texas Medical Branch, Korea University, and Southern Medical University. Finally, the dark taupe cluster consists of the University of Wisconsin-Madison, the University of Florida, and the University of Iowa.

These findings highlight the leading institutions and their collaborative networks in placental oxidative stress research, providing valuable insights into the global research landscape and identifying potential opportunities for future collaborations.

#### 3.3.2 Author distribution

Using the bibliometrix package in R, we analyzed author distribution in placental oxidative stress research. Graham J. Burton from the University of Cambridge emerged as the most prolific author with 73 publications, followed by Eric Jauniaux from University College London with 47 publications, and Babbette LaMarca from the University of Mississippi with 45 publications (Table 3). Graham J. Burton also had the highest average citations and H-index among the top authors.

**TABLE 3:** Top 10 most productive authors in the Field of Placental Oxidative Stress.

Rank	Author	Institution	Publications n (%)	Total citations	Average citations	H-index
1	Burton, Graham J	University of Cambridge	73 (1.52)	11,182	153.18	51
2	Jauniaux, Eric	University College London	47 (0.98)	6959	148.06	31
3	LaMarca, Babbette	University of Mississippi	45 (0.94)	1985	44.11	24
4	Menon, Ramkumar	University of Texas Medical Branch Galveston	42 (0.88)	1721	40.98	23
5	Perkins, Anthony V	Griffith University	36 (0.75)	1261	35.03	22
6	Myatt, Leslie	Oregon Health and Science University	35 (0.73)	3911	111.74	27
7	Cornelius, Denise C	University of Mississippi Medical Center	31 (0.65)	1163	37.52	16
8	Qi, Hongbo	Chongqing Medical University	29 (0.6)	520	17.93	14
9	Amaral, Lorena M	University of Mississippi	28 (0.58)	1153	41.18	14
10	Mitsumori, Kunitoshi	Tokyo University of Agriculture and Technology	28 (0.58)	500	17.86	14

**FIGURE 4:**
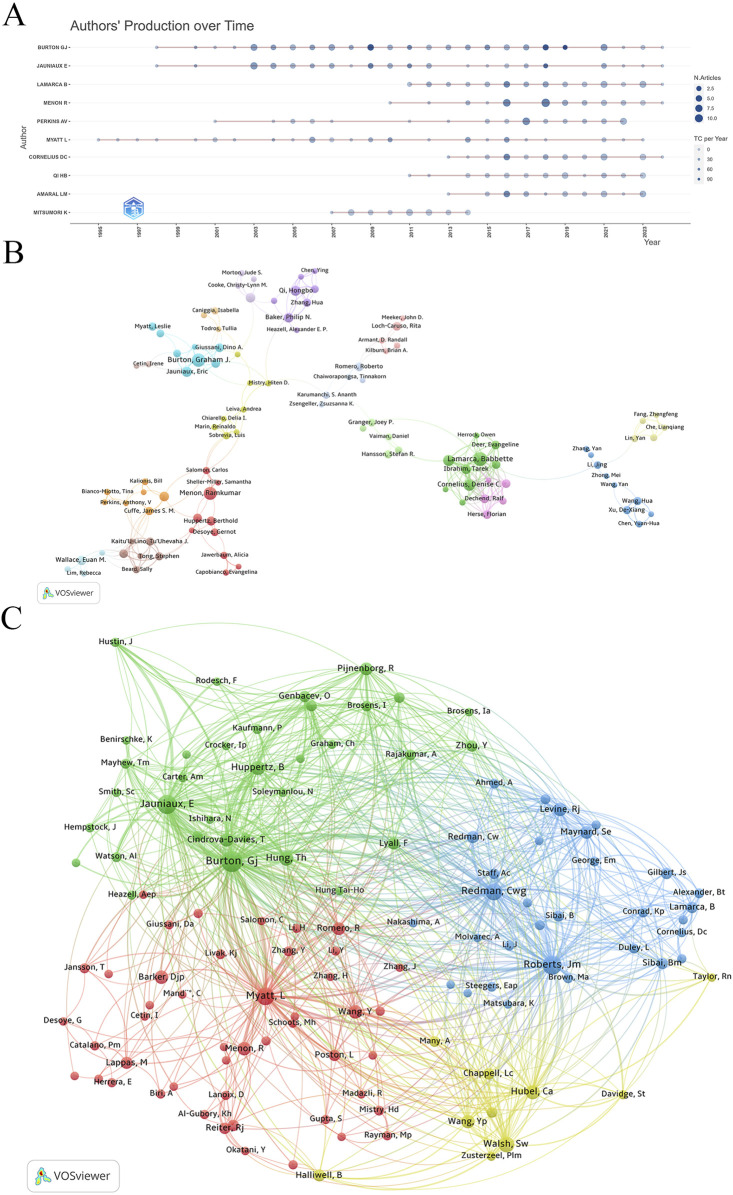
Collaboration network of authors. **(A)** Authors’ production over time. **(B)** Visualization map of author collaboration. **(C)** Co-citation analysis of cited authors.

Analyzing authors’ annual research output revealed that Graham J. Burton, Eric Jauniaux, and Leslie Myatt have consistently contributed to the field over the past 2 decades (Figure 4A). Recent years have seen increased activity from Denise C. Cornelius, Hongbo Qi, and Lorena M. Amaral, indicating significant progress in specific research areas.

Using VOSviewer for co-authorship analysis, we set the parameters to “association strength” with a minimum document threshold of 8. Out of 167 qualifying authors, VOSviewer classified them into clusters based on co-authorship frequency and density (Figure 4B). Graham J. Burton has close collaborative ties with Eric Jauniaux, Tereza Cindrova-Davies, Dino A. Giussani, Leslie Myatt, and D. Stephen Charnock-Jones. Another network includes Babbette LaMarca, Denise C. Cornelius, Lorena M. Amaral, Tarek Ibrahim, and Nathan Campbell. Additionally, Anthony V. Perkins collaborates with James S. M. Cuffe, Bill Kalionis, Tina Bianco-Miotto, Jing Li, and Shaun P. Brennecke.

Co-citation analysis identified Graham J. Burton, Eric Jauniaux, James M. Roberts, Leslie Myatt, and Christopher W. Redman as the five most frequently cited authors, highlighting their central roles and substantial influence in the research network (Figure 4C). Their citation counts significantly surpass those of other authors, underscoring their key positions in the field of placental oxidative stress.

### 3.4 Subject and journal distribution

#### 3.4.1 Subjects

The analysis of publication volume identified Obstetrics and Gynecology, Reproductive Biology, and Biochemistry and Molecular Biology as the top three subjects in placental oxidative stress research (Table 4). Additional key subjects included Developmental Biology, Cell Biology, and Toxicology. These results demonstrated a strong focus on medical and biological disciplines, highlighting the interdisciplinary nature of placental oxidative stress studies and their broad implications for maternal and fetal health.

**TABLE 4:** Top 10 subject categories in the field of Placental Oxidative Stress.

Rank	Web of science categories	Publications n (%)
1	Obstetrics Gynecology	973 (20.288)
2	Reproductive Biology	805 (16.785)
3	Biochemistry Molecular Biology	708 (14.762)
4	Developmental Biology	469 (9.779)
5	Cell Biology	424 (8.841)
6	Toxicology	398 (8.299)
7	Endocrinology Metabolism	360 (7.506)
8	Pharmacology Pharmacy	321 (6.693)
9	Medicine Research Experimental	288 (6.005)
10	Physiology	281 (5.859)

#### 3.4.2 Journal distribution

An in-depth analysis of journal distribution was conducted using the bibliometrix package in R, identifying 1,173 journals publishing relevant articles. The top 10 journals with the highest number of publications on “placental oxidative stress” are led by *Placenta*, with 329 articles, accounting for 6.86% of the total publications (Table 5). *Placenta* has accumulated 17,152 citations and an H-index of 67, underscoring its significant impact and authoritative status in obstetrics and gynecology. The International Journal of Molecular Sciences follows with 122 publications. Other notable journals include *PLOS One*, *Reproductive Sciences*, and *Antioxidants*, which also exhibit substantial publication volumes and academic influence. The *American Journal of Obstetrics and Gynecology*, although not at the forefront in publication volume, ranks highly in average citations and has an impact factor of 8.7, reflecting its prominent position in the field.

**TABLE 5:** Top 10 journals in the field of Placental Oxidative Stress.

Journal	Publications n (%)	Total citations	Average citations	H-index	JCR category	Category quartile	Journal impact factor (2023)
Placenta	329 (6.86)	17,152	52.13	67	Obstetrics and Gynecology	Q1	3
International Journal of Molecular Sciences	122 (2.54)	3067	25.14	29	Biochemistry and Molecular Biology	Q1	4.9
Plos One	89 (1.86)	2904	32.63	31	Multidisciplinary Sciences	Q1	2.9
Reproductive Sciences	71 (1.48)	1158	16.31	20	Obstetrics and Gynecology	Q2	2.6
Antioxidants	67 (1.40)	518	7.73	13	Biochemistry and Molecular Biology	Q1	6
Scientific Reports	63 (1.31)	1513	24.02	23	Multidisciplinary Sciences	Q1	3.8
American Journal of Obstetrics and Gynecology	61 (1.27)	4585	75.16	39	Obstetrics and Gynecology	Q1	8.7
Journal of Maternal-Fetal and Neonatal Medicine	59 (1.23)	1517	25.71	20	Obstetrics and Gynecology	Q1	1.7
Biology of Reproduction	52 (1.09)	1854	35.65	27	Reproductive Biology	Q1	3.1
Free Radical Biology and Medicine	47 (0.98)	2344	49.87	24	Biochemistry and Molecular Biology	Q1	7.1

**FIGURE 5:**
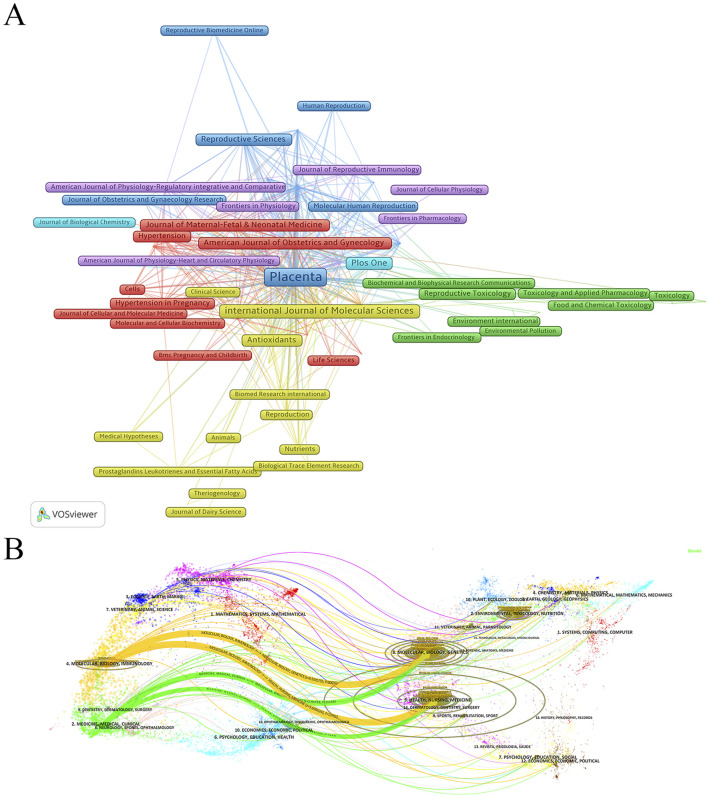
Analysis of journal sources. **(A)** Co-citation analysis of cited sources. **(B)** Dual-Map overlay of journals publishing research on Placental Oxidative Stress.

A co-citation analysis using VOSviewer was conducted to analyze journal relationships (Figure 5A). In the resulting visualization, journals were represented by nodes, and lines illustrated co-citation relationships. Node size corresponded to the number of publications, while the thickness of connecting lines reflected the strength of associations between journals. Stronger connections, indicated by thicker lines, suggested more frequent co-citations and higher similarities in research topics and methodologies.

Additionally, a dual-map overlay of journals generated in CiteSpace revealed key details about journal relationships and citation patterns. The left side of the overlay represented citing journals, while the right side showed cited journals, with subject areas and citation paths clearly identified. Two prominent citation pathways, distinguished by orange and green hues, highlighted the prevalent citations extending from journals in fields such as molecular/biology/genetics and health/nursing/medicine, to those in domains like molecular biology/immunology and medicine/clinical medicine (Figure 5B). These findings offered valuable insights into the flow of knowledge and academic influence across disciplines, underscoring the interdisciplinary nature and wide-reaching impact of placental oxidative stress research.

#### 3.4.3 Top 10 highly cited and co-cited articles

**FIGURE 6:**
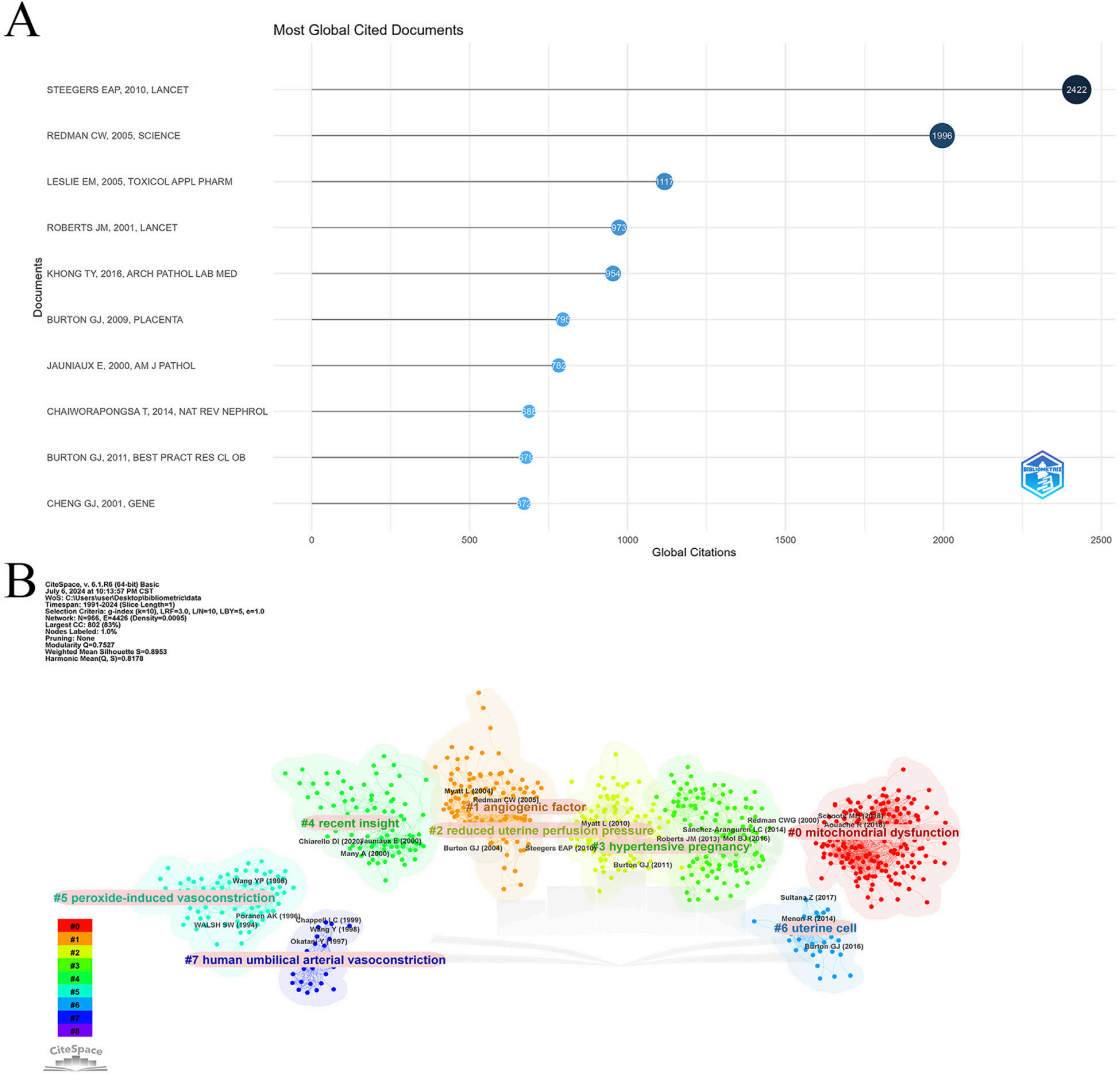
Highly cited and co-cited references. **(A)** Top 10 most cited articles in Placental oxidative stress. **(B)** Cluster analysis of co-cited references.

Using the bibliometrix package in R, a comprehensive analysis of cited and co-cited references was conducted, encompassing 4,176 articles with a total of 177,497 citations and a median citation count of 16. Table 6 and Figure 6A detail the top 10 most cited articles, with the 2010 Lancet article “Pre-eclampsia” leading with 2,422 citations and the highest Normalized Global Citations index of 29.71.

**TABLE 6:** Top 10 most cited references in the field of Placental Oxidative Stress.

Title	Journal	Year of publication	Total Citations	Normalized global citations
Pre-eclampsia	Lancet	2010	2422	29.71
Latest advances in understanding preeclampsia	Science	2005	1996	19.38
Multidrug resistance proteins: role of P-glycoprotein, MRP1, MRP2, and BCRP (ABCG2) in tissue defense	Toxicology and Applied Pharmacology	2005	1117	10.84
Pathogenesis and genetics of pre-eclampsia	Lancet	2001	973	9.43
Sampling and Definitions of Placental Lesions Amsterdam Placental Workshop Group Consensus Statement	Archives of Pathology and Laboratory Medicine	2016	954	24.19
Rheological and Physiological Consequences of Conversion of the Maternal Spiral Arteries for Uteroplacental Blood Flow during Human Pregnancy	Placenta	2009	795	10.39
Onset of maternal arterial blood flow and placental oxidative stress - A possible factor in human early pregnancy failure	American Journal of Pathology	2000	782	10.08
Pre-eclampsia part 1: current understanding of its pathophysiology	Nature Reviews Nephrology	2014	688	13.99
Oxidative stress	Best Practice and Research Clinical Obstetrics and Gynaecology	2011	679	12.55
Homologs of gp91phox: cloning and tissue expression of Nox3, Nox4, and Nox5	Gene	2001	672	6.52

Table 7 lists the top 10 most frequently co-cited references. The article “Oxidative stress in the placenta” is the most co-cited with 330 mentions, followed by “Onset of maternal arterial blood flow and placental oxidative stress” with 320 co-citations, and “Excess placental soluble fms-like tyrosine kinase 1 (sFlt1)” with 299 co-citations. Notably, “Latest advances in understanding preeclampsia” and “Onset of maternal arterial blood flow and placental oxidative stress” are among both the top cited and co-cited references, indicating their foundational impact in the field.

**TABLE 7:** Top 10 most co-cited references in the field of Placental Oxidative Stress.

Rank	Title	Journal	Year of publication	Number of references co-cited
1	Oxidative stress in the placenta	Histochemistry and Cell Biology	2004	330
2	Onset of maternal arterial blood flow and placental oxidative stress - A possible factor in human early pregnancy failure	The American Journal of Pathology	2000	320
3	Excess placental soluble fms-like tyrosine kinase 1 (sFlt1) may contribute to endothelial dysfunction, hypertension, and proteinuria in preeclampsia	The Journal of Clinical Investigation	2003	299
4	Latest advances in understanding preeclampsia	Science	2005	232
5	Placental oxidative stress: From miscarriage to preeclampsia	Journal of the Society for Gynecologic Investigation	2004	229
6	Circulating angiogenic factors and the risk of preeclampsia	The New England Journal of Medicine	2004	221
7	Oxidative stress in the pathogenesis of preeclampsia	Proceedings of the Society for Experimental Biology and Medicine	1999	203
8	Protein measurement with the Folin phenol reagent	The Journal of Biological Chemistry	1951	192
9	Pre-eclampsia	Lancet	2005	181
10	Trophoblastic oxidative stress in relation to temporal and regional differences in maternal placental blood flow in normal and abnormal early pregnancies	The American Journal of Pathology	2003	176

CiteSpace was employed to analyze co-citation relationships (Figure 6B), revealing a network of 966 nodes and 4,426 links, organized into eight major clusters: mitochondrial dysfunction, angiogenic factors, reduced uterine perfusion pressure, hypertensive pregnancy, recent insights, peroxide-induced vasoconstriction, uterine cells, and human umbilical arterial vasoconstriction. This analysis highlights the diverse research themes within placental oxidative stress and emphasizes key areas of ongoing investigation.

### 3.5 Keyword analysis

#### 3.5.1 Keyword distribution and co-occurrence analysis

**FIGURE 7:**
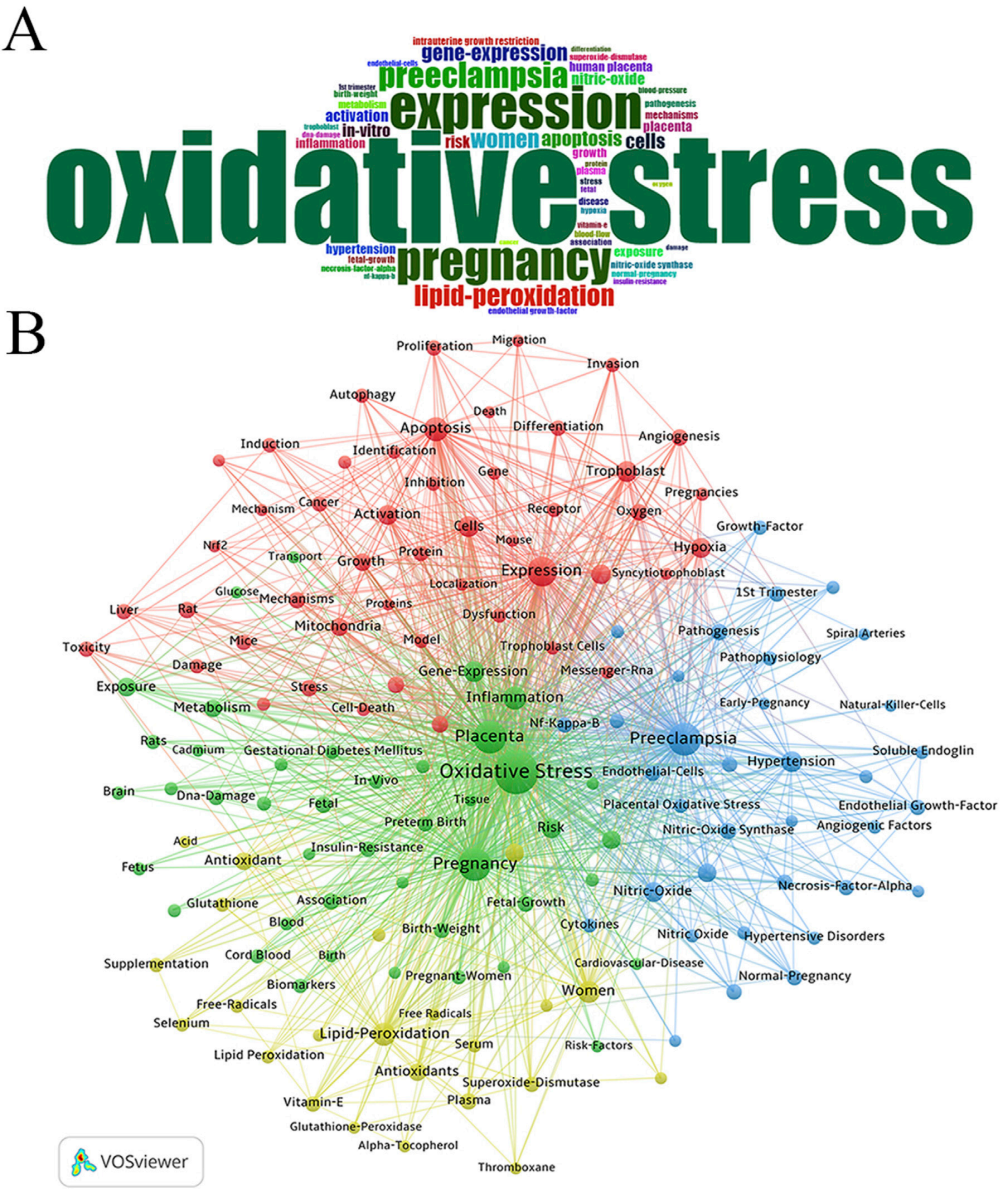
Analysis of keywords associated with Placental Oxidative Stress. **(A)** Word cloud analysis. **(B)** Clustering of keywords.

The word cloud analysis using the bibliometrix package (Figure 7A) revealed the distribution of keywords, with frequent terms including “oxidative stress,” “expression,” “pregnancy,” “preeclampsia,” “lipid peroxidation,” and “gene expression,” highlighting central research themes in the field. Further analysis using VOSviewer, applying the association strength method with a minimum keyword occurrence threshold of 50, identified 149 relevant keywords from a dataset of 15,440 (Figure 7B). The co-occurrence network showed “Oxidative Stress” as the most frequently mentioned keyword, followed by “Pregnancy” and “Preeclampsia.”

Four main clusters emerged among the top 50 keywords:

Green Cluster: Led by “Oxidative Stress” and including related terms such as Pregnancy, Placenta, Inflammation, Gene Expression, Risk, Intrauterine Growth Restriction, Exposure, and Metabolism.

Blue Cluster: Focused on terms like Preeclampsia, Hypertension, Nitric Oxide, Nitric Oxide Synthase, Pathogenesis, Endothelial Growth Factor, Normal Pregnancy, and Blood Flow. Red Cluster: Centered on terms such as Expression, Apoptosis, Cells, Trophoblast, *In Vitro*, Hypoxia, Activation, Growth, Mitochondria, Mechanisms, Reactive Oxygen Species, and Stress. Yellow Cluster: Highlighted keywords like Lipid Peroxidation, Women, Human Placenta, Antioxidants, Plasma, Superoxide Dismutase, Vitamin E, and Glutathione.

The analysis emphasized “Oxidative Stress” as a central theme in the field, with strong connections to other key concepts such as pregnancy complications and cellular mechanisms. The cluster distribution reflected major research interests and their interconnections.

#### 3.5.2 Burst detection analysis

**FIGURE 8:**
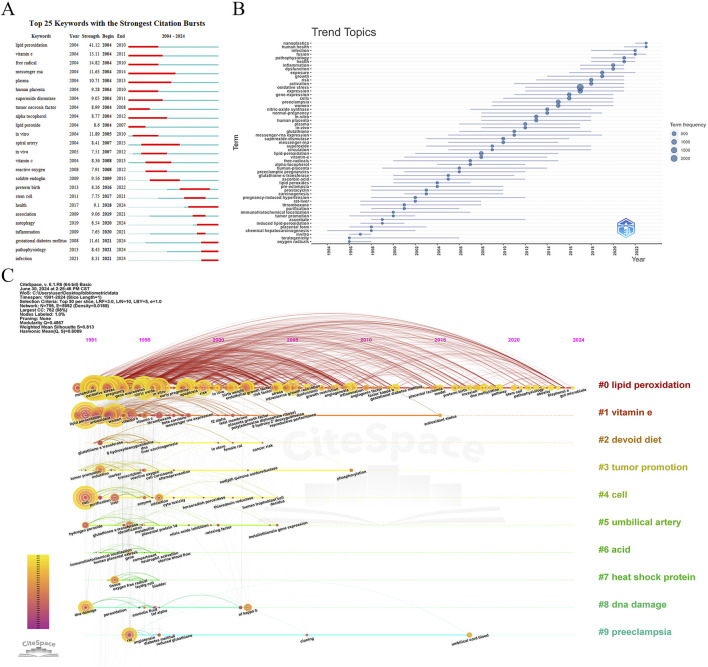
Keyword analysis in Placental Oxidative Stress. **(A)** Burst detection of keywords. **(B)** Trend topics analysis. **(C)** A timeline visualization in CiteSpace.

The burst detection analysis of keywords over the past 20 years revealed significant frequency increases, highlighting emerging trends in the field. Figure 8A presents the top 25 keywords with the highest burst strength. “Lipid peroxidation” had the strongest burst from 2004 to 2010, followed by key terms such as “vitamin E,” “free radical,” “*in vitro*,” “messenger RNA,” “gestational diabetes mellitus,” “plasma,” “soluble endoglin,” “human placenta,” and “health.” Keywords like “lipid peroxidation,” “vitamin E,” and “free radical” gained prominence over the past decade, while recent surges in “gestational diabetes mellitus,” “autophagy,” and “pathophysiology” indicate emerging research interests.

#### 3.5.3 Trend topics analysis

The trend topics analysis conducted using the bibliometrix package provided insights into the evolution of keyword and subject trends over time. Figure 8B outlined key developments:

1994-1998: Early research focused on “oxygen radicals,” “teratogenicity,” and “*in vitro*,” with less emphasis on “placental form” and “chemical hepatocarcinogenesis.”

1998-2008: Emerging themes included “ascorbate,” “induced lipid-peroxidation,” and “immunohistochemical localization,” alongside persistent topics like “pregnancy-induced hypertension,” “rat liver,” and “prostacyclin.”

2008-Present: Significant growth occurred in keywords such as “lipid peroxidation,” “vitamin E,” and “superoxide,” with “oxidative stress” and “expression” emerging as central themes. Established topics like “pregnancy-induced hypertension,” “carcinogenesis,” and “free radicals” remained relevant.

The evolution of research topics reflected a shift from foundational studies to a more detailed focus on biochemical processes and their implications. The increasing research on “lipid peroxidation,” “vitamin E,” and “superoxide” underscored their current importance.

#### 3.5.4 A timeline visualization in CiteSpace

The CiteSpace timeline view provides an overview of the evolution of research topics in “placental oxidative stress” from 1991 to 2024. Using a 1-year time slice and focusing on the top 50 keywords, the analysis generated a network with 796 nodes and 5,992 links (density = 0.0189) (Figure 8C). The top 10 clusters identified were: Lipid Peroxidation, Vitamin E, Devoid Diet, Tumor Promotion, Cell, Umbilical Artery, Acid, Heat Shock Protein, DNA Damage and Preeclampsia.

The timeline view highlighted key research themes and their progression over time. Early keywords (1991-2000) include “oxidative stress,” “expression,” “pregnancy,” “preeclampsia,” and “lipid peroxidation,” reflecting foundational research topics. Recent keywords (2020-2024) such as “pathophysiology,” “obesity,” “outcome,” and “air pollution” indicate emerging areas of focus and new challenges in the field.

## 4 Discussion

This bibliometric study employs CiteSpace, VOSviewer, and the Bibliometrix package to perform an in-depth analysis of the literature related to “placental oxidative stress,” providing a comprehensive overview of the field’s research outputs and advancements. We conducted a quantitative analysis of annual publication volumes, country distributions, research institutions, author contributions, interdisciplinary interactions, journal distributions, and keywords.

Among the top ten authors, Professor Graham J. Burton and Eric Jauniaux have forged a close collaborative relationship, offering novel and profound insights into the mechanisms governing placental formation and function during early pregnancy. Their joint research has significantly expanded our understanding of how the placenta supports fetal growth and development (Burton and Jauniaux, 2023; Phengpol et al., 2023; Zhang et al., 2023a; Hu et al., 2020; Fortis et al., 2018; Anto et al., 2023; Amaral et al., 2013). Specifically, their systematic investigations have revealed the pathophysiological basis of pregnancy complications such as preeclampsia (Phengpol et al., 2023; Zhang et al., 2023a; Hu et al., 2020; Fortis et al., 2018; Anto et al., 2023; Amaral et al., 2013; Burton et al., 2019), gestational diabetes mellitus (GDM) (Phengpol et al., 2023; Zhang et al., 2023a; Hu et al., 2020; Fortis et al., 2018; Anto et al., 2023; Amaral et al., 2013; Ferreira et al., 2023), and fetal growth restriction (Phengpol et al., 2023; Zhang et al., 2023a; Hu et al., 2020; Burton and Jauniaux, 2018; Fortis et al., 2018; Anto et al., 2023; Amaral et al., 2013). These findings address key pathological pathways including oxidative stress (Phengpol et al., 2023; Zhang et al., 2023a; Hu et al., 2020; Fortis et al., 2018; Anto et al., 2023; Amaral et al., 2013; Jauniaux and Burton, 2016; Cindrova-Davies et al., 2018), endoplasmic reticulum stress (Phengpol et al., 2023; Zhang et al., 2023a; Hu et al., 2020; Fortis et al., 2018; Anto et al., 2023; Amaral et al., 2013; Burton and Yung, 2011), and mitochondrial dysfunction (Phengpol et al., 2023; Zhang et al., 2023a; Hu et al., 2020; Fortis et al., 2018; Anto et al., 2023; Amaral et al., 2013; Yung et al., 2019), opening new avenues for prevention and treatment strategies. Their research also highlights the potential adverse impacts of environmental factors, including air pollution (Phengpol et al., 2023; Zhang et al., 2023a; Hu et al., 2020; Fortis et al., 2018; Anto et al., 2023; Amaral et al., 2013; Fussell et al., 2024; Bearblock et al., 2021), hypoxic conditions, and high-altitude exposure (Phengpol et al., 2023; Zhang et al., 2023a; Hu et al., 2020; Fortis et al., 2018; Anto et al., 2023; Amaral et al., 2013; Kurlak et al., 2016; Tissot et al., 2010) on placental function and pregnancy outcomes, significantly raising awareness about the link between environmental health risks and pregnancy outcomes. Methodologically, Professors Burton and Jauniaux have utilized a range of modern biological tools, including RNA-Seq transcriptome sequencing (Phengpol et al., 2023; Zhang et al., 2023a; Hu et al., 2020; Fortis et al., 2018; Anto et al., 2023; Amaral et al., 2013; Prater et al., 2021), digital PCR for high-precision quantification (Hu et al., 2020; Fortis et al., 2018; Anto et al., 2023; Amaral et al., 2013; Allerkamp et al., 2021), and advanced ultrasound imaging techniques (Hu et al., 2020; Fortis et al., 2018; Anto et al., 2023; Amaral et al., 2013; Jauniaux et al., 2005). The integration of these technologies has greatly deepened our understanding of the molecular mechanisms underlying placental physiology and pathology while driving innovations in placental research techniques. Overall, the research by Professor Burton and Jauniaux spans multiple disciplines including biology, medicine, and environmental science, promoting deep communication and interdisciplinary collaboration, and injecting new vitality into the comprehensive development of placental science. Their work has made significant contributions to improving maternal and infant health globally.

Professor Babbette LaMarca’s research provides a profound analysis of gestational hypertension, particularly preeclampsia (Fortis et al., 2018; Anto et al., 2023; Amaral et al., 2013; Deer et al., 2023), emphasizing the central role of immune cell subpopulations such as T cells (Fortis et al., 2018; Anto et al., 2023; Amaral et al., 2013; Hogg et al., 2024; Deer et al., 2021a), B cells (Fortis et al., 2018; Anto et al., 2023; Amaral et al., 2013; Herrock et al., 2023), and natural killer cells (Fortis et al., 2018; Anto et al., 2023; Amaral et al., 2013; Cunningham et al., 2020), as well as their released cytokines [e.g., IL-17 (Fortis et al., 2018; Anto et al., 2023; Fitzgerald et al., 2023), TNF-α (Cunningham et al., 2020), AT1-AA (Herrock et al., 2023)] in the initiation and progression of the disease. Her work further explores the complex associations between key pathophysiological processes such as placental ischemia (Bakrania et al., 2020), endothelial dysfunction (Deer et al., 2021b), and mitochondrial oxidative stress (Cunningham et al., 2020), with gestational hypertension. Notably, Professor LaMarca focuses on mitochondrial dysfunction (Deer et al., 2021c) and oxidative stress (Vaka et al., 2022) as critical mediators of gestational hypertension and its severe complications (e.g., multi-organ dysfunction), revealing their central role in the disease process. Based on this, she has investigated potential strategies for treating or alleviating gestational hypertension through targeting mitochondrial function (Vaka et al., 2021) and oxidative stress (Deer et al., 2021b). Particularly noteworthy is her extensive discussion of AT1-AA as a core molecule, with its role in mediating gestational hypertension and related pathophysiological changes detailed in multiple papers. This has led to new therapeutic approaches involving the blockade of the AT1-AA pathway to improve disease conditions and related complications (Hogg et al., 2024; Herrock et al., 2023; Cunningham et al., 2018). Additionally, Professor LaMarca’s research utilizes animal models to simulate gestational hypertension and its pathophysiological changes (Cornelius et al., 2015), providing an important platform for understanding disease mechanisms and evaluating treatment interventions. Through various experiments, the study assesses the potential effects of several therapeutic strategies, including vitamin D supplementation (Faulkner et al., 2016), IL-10 supplementation (Harmon et al., 2015), and magnesium sulfate treatment (Johnson et al., 2014), offering valuable insights for clinical practice. In summary, Professor LaMarca’s research not only deepens our understanding of the pathogenesis of gestational hypertension but also opens new scientific pathways and directions for the prevention and treatment of this disease, with significant theoretical and practical implications.

Professor Ramkumar Menon extensively explored the interactions at the maternal-fetal interface under various physiological and pathological conditions, including normal pregnancy, preterm birth (Menon, 2022), and preterm premature rupture of membranes (Mikkelsen et al., 2023). His research emphasized the roles of extracellular vesicles (EVs) at the maternal-fetal interface, particularly in transmembrane signaling molecule transfer (Kalia et al., 2023), modulation of local and systemic inflammatory responses (Shepherd et al., 2021), and potential involvement in drug transport across the maternal-fetal barrier (Sheller-Miller et al., 2016). These findings expanded the understanding of molecular communication mechanisms at the maternal-fetal interface and laid the foundation for developing EV-based diagnostic and therapeutic strategies. Professor Menon also elucidated key mechanisms through which oxidative stress contributes to pregnancy complications, emphasizing its role in driving conditions such as preterm birth and premature rupture of membranes (Arita et al., 2019; Jin et al., 2018). His work provided critical insights into the biological mechanisms underlying these complex pregnancy issues. On a technical level, Professor Menon introduced innovative technologies into pregnancy research, including organ-on-chip technology to simulate *in vivo* microenvironments (Vidal et al., 2024), microfluidic systems to optimize drug testing and pharmacokinetic analysis (Kammala et al., 2023), and quantitative proteomics for analyzing changes in protein expression at the maternal-fetal interface (Menon et al., 2019). These advancements enhanced the precision and reproducibility of research, offering powerful tools for investigating the complex physiological and pathological processes in pregnancy, thereby advancing the field of pregnancy science.

Professor Anthony V. Perkins extensively investigated the dynamic changes in placental mitochondria throughout pregnancy (Bartho et al., 2020; Fisher et al., 2020; Holland et al., 2018), including pathological conditions such as gestational diabetes (Fisher et al., 2021) and preeclampsia (Holland et al., 2018). He systematically evaluated the significant impacts of these changes on pregnancy outcomes, (Cuffe JS. et al., 2017; Perkins, 2011), focusing on how oxidative stress contributes to complications by impairing placental function (Habibi et al., 2021a). In addition to these findings, Professor Perkins concentrated on nutritional interventions, particularly selenium (Hogan and Perkins, 2022) and iodine supplementation (Habibi et al., 2021a), assessing their potential to counteract the harmful effects of oxidative stress on the placenta and fetus. Through a series of well-designed studies, he demonstrated that appropriate trace element supplementation enhances the placenta’s antioxidant defenses (Habibi et al., 2021b), supporting normal placental function and fetal development (Hogan and Perkins, 2022). His research emphasized the importance of adequate trace element supplementation, including selenium, iodine, and iron, in optimizing placental function (Richard et al., 2017), promoting fetal growth, and improving pregnancy outcomes (Hofstee et al., 2019). Furthermore, Professor Perkins explored the application of placental-derived biomarkers for the early prediction and diagnosis of pregnancy complications (Cuffe JS. et al., 2017). By analyzing the patterns of these biomarkers and their associations with pregnancy outcomes, he advanced early detection and intervention strategies (Cuffe J. et al., 2017), contributing significantly to maternal and infant health research.

The top ten highly-cited articles in “placental oxidative stress” showcase advancements and deeper insights in the field. Several articles concentrated on the pathophysiology of preeclampsia (Roberts and Cooper, 2001; Steegers et al., 2010; Redman and Sargent, 2005), examining its mechanisms, genetic underpinnings, and recent developments, underscoring the prominence of this condition in related research. Other key studies explored the role of multidrug resistance proteins in tissue defense (Leslie et al., 2005), the sampling and classification of placental lesions (Khong et al., 2016), and the rheological and physiological effects of maternal spiral artery remodeling on uterine-placental blood flow (Burton et al., 2009). Research into oxidative stress mechanisms (Burton and Jauniaux, 2011; Jauniaux et al., 2000) and the cloning and tissue expression of Nox family homologs (Cheng et al., 2001) also demonstrated the breadth and depth of inquiry in the field. These highly-cited studies not only highlight current research priorities but also provide essential references for future directions.

Frequently cited references among these articles focus on the role of placental oxidative stress in pathological processes such as pregnancy failure (Jauniaux et al., 2000; Burton and Jauniaux, 2004) and preeclampsia (Hubel, 1999; Sibai et al., 2005), marking oxidative stress as a critical research area. In-depth analyses of preeclampsia, including the excessive expression of soluble fms-like tyrosine kinase one in its pathogenesis (Maynard et al., 2003) and the involvement of circulating angiogenic factors (Levine et al., 2004), reveal the complexity and scope of research in the domain. Additionally, references related to protein measurement methods (Lowry et al., 1951) and the connection between spatiotemporal variations in placental blood flow and oxidative stress (Jauniaux et al., 2003) offer vital technical support and theoretical guidance for ongoing research. These widely cited references lay the groundwork for the field and provide valuable insights for future investigations and clinical intervention strategies.

In the VOSviewer keyword co-occurrence network analysis, frequent joint appearances of keywords within clusters indicated their significant role in shaping the content of the field. Four color-coded clusters emerged from the analysis:

The green cluster revealed the broad impact of “Oxidative Stress” on pregnancy, placental function, and fetal development, with a particularly prominent focus (Aouache et al., 2018; Cheong et al., 2016). Keywords such as “Pregnancy” and “Placenta” pointed to the central role of oxidative stress in these physiological processes. Close associations among keywords like “Inflammation,” “Gene-Expression,” and “Risk” provided deeper insights into oxidative stress mechanisms and underscored its pivotal role in various pregnancy complications.

Oxidative stress contributed significantly to the pathogenesis of conditions such as preeclampsia (Tenorio et al., 2019), GDM (Sha et al., 2019), and fetal growth restriction (Moon et al., 2021) through inflammatory factor activation. These disruptions in placental function involved excessive inflammatory cytokine release (Bernardi et al., 2012), reduced oxidase activity (Mou et al., 2020), and elevated oxidative stress biomarkers (Zhou et al., 2019). Maternal factors such as overweight (Phengpol et al., 2023), obesity (Liang et al., 2018), environmental pollutant exposure (Huang et al., 2017; Riggs et al., 2020), and nutritional deficiencies (Dominguez-Perles et al., 2019) exacerbated oxidative stress and inflammation, further promoting pregnancy complications. These findings emphasized the importance of managing environmental and lifestyle factors during pregnancy. Moreover, certain natural compounds [e.g., astaxanthin (Xuan et al., 2016), pomegranate polyphenols (Chen et al., 2023), and mangiferin (Sha et al., 2019)] and medications [e.g., vitamin D (Nunes et al., 2022), magnesium sulfate (Han et al., 2018), and folic acid (Zhang et al., 2023a)] showed potential in reducing oxidative stress and inflammation, offering promising therapeutic strategies for clinical intervention.

The green cluster also highlighted dynamic changes in gene expression profiles during pregnancy, tightly linked to oxidative stress. Oxidative stress regulated specific genes, such as phospholipase A2 (Brien et al., 2017) and GPX1 (Endler et al., 2016), and indirectly influenced gene activity through epigenetic modifications like DNA methylation (Garcia-Contreras et al., 2019), affecting pregnancy outcomes and offspring health. Environmental factors [e.g., Wi-Fi radiation (Vafaei et al., 2020), maternal obesity (McCoski et al., 2018)], physiological changes (Miyagami et al., 2013), nutritional supplementation (Shorey-Kendrick et al., 2021), and assisted reproductive technologies (Zhang et al., 2010) all impacted gene expression in the placenta and fetus, presenting new perspectives for understanding pregnancy pathophysiology.

Oxidative stress posed a significant risk in assisted reproductive technologies (Mauchart et al., 2023), calling for antioxidant strategies to improve safety. Polymorphisms in DNA repair genes, such as APE1 and XRCC1, were closely linked to heightened risks of preeclampsia (Vural et al., 2009) and preterm birth, underscoring the genetic component in risk assessment. Exposure to pollutants like microplastics (Liu et al., 2023), heavy metals (Shachar et al., 2013), and organophosphate flame retardants (Li et al., 2023) further increased the risk of pregnancy complications and offspring health issues, highlighting the need for environmental protection. Maternal nutritional deficiencies, such as those in vitamin D (Fondjo et al., 2024) and selenium (Lewandowska et al., 2019) along with adverse lifestyle habits like smoking (England and Zhang, 2007) and physical inactivity (Huang et al., 2019), elevated the risks of preeclampsia, preterm birth, and metabolic syndrome. These findings underscored the necessity of multifactorial assessments in predicting and managing pregnancy risks, with future research focused on exploring the mechanisms of these interactions to develop more precise preventive and intervention strategies.

The green cluster included keywords related to fetal growth and development, such as “Intrauterine Growth Restriction,” “Fetal Growth,” “Birth Weight,” and “Preterm Birth.” These terms reflected the significant impact of oxidative stress on intrauterine growth patterns (Yoshida et al., 2018) and fetal birth weight (Hu et al., 2020), highlighting its role as a major risk factor for adverse pregnancy outcomes, including preterm birth (Menon, 2014; Stefanovic et al., 2019). Oxidative stress affects placental function through various mechanisms, negatively impacting fetal growth and development (Luo et al., 2018; Matsubara and Sato, 2001; Jones et al., 2013). These mechanisms include the regulation of mitochondrial content and cell cycle progression, influencing placental development (Luo et al., 2018), as well as the production of large amounts of ROS by enzymes like NAD(P)H oxidase, which elevate oxidative stress levels in the placenta (Matsubara and Sato, 2001). Additionally, changes in nitric oxide synthase activity further modulate oxidative stress, affecting the development of organs such as the fetal kidneys (Figueroa et al., 2016). Maternal nutrition also plays a critical role in fetal growth. While the intake of ω-3 fatty acids may enhance placental antioxidant capacity, it does not effectively prevent IUGR caused by placental ischemia-reperfusion injury (Jones et al., 2013). In contrast, maternal supplementation with nutrients like folic acid can alleviate IUGR induced by high-fat diets by reducing placental inflammation and oxidative stress (Zhang et al., 2023a). Research on the relationship between IUGR and oxidative stress has revealed a strong correlation through the detection of oxidative stress markers in placental tissue and fetal serum. Elevated levels of lipid peroxidation products, such as malondialdehyde, in IUGR fetal serum, along with decreased activity of antioxidant enzymes like superoxide dismutase, have been observed (Llanos and Ronco, 2009). Furthermore, increased levels of heavy metals in the placenta suggest that environmental pollutants may impair fetal growth by inducing oxidative stress (Llanos and Ronco, 2009). Oxidative stress not only affects fetal development but also has significant implications for maternal health. For instance, IUGR pregnancies are often associated with endothelial dysfunction and elevated systemic oxidative stress, which may contribute to the occurrence of IUGR (Yoshida et al., 2018). Additionally, imbalances in angiogenic factors and oxidative stress biomarkers have been noted in older pregnant women, potentially linking these factors to adverse pregnancy outcomes (Odame et al., 2018). Various antioxidant interventions have been shown to effectively reduce oxidative stress and improve fetal growth in IUGR cases. Antioxidants such as N-acetylcysteine, hydroxychloroquine (Dai et al., 2024), and melatonin (Alers et al., 2013; Miller et al., 2014) have demonstrated efficacy in mitigating oxidative stress and autophagy levels, promoting healthier fetal development. Autophagy, a cellular protective mechanism, has also been found to play a vital role in reducing placental apoptosis and oxidative stress (Zhang et al., 2023b).

Oxidative stress significantly contributes to low birth weight. In malnourished pregnancies, maternal supplementation with melatonin enhances placental efficiency and birth weight by upregulating antioxidant enzyme expression in the placenta, thereby safeguarding placental function and fostering fetal development (Richter et al., 2009). This underscores the potential of antioxidants in ameliorating adverse pregnancy outcomes. Additionally, research indicates that as maternal body mass index increases, nitrative stress levels in the placenta markedly rise, whereas oxidative stress levels may not exhibit a corresponding increase and may even decline in some instances (Roberts et al., 2009). This observation suggests a potential balance between nitrative and oxidative stress in obese pregnancies, potentially serving as a protective mechanism for the placenta. Additionally, research has focused on placental function in IUGR and low birth weight fetuses, revealing that these placentas are more prone to oxidative damage, mitochondrial dysfunction, and impaired angiogenesis (Hu et al., 2020). These findings further emphasize the crucial role of oxidative stress in the occurrence of IUGR and low birth weight. Notably, exposure to certain environmental pollutants, such as trichloroethylene, has been associated with elevated placental oxidative stress, contributing to fetal growth restriction (Loch-Caruso et al., 2019). These discoveries underscore the importance of regulating oxidative stress to maintain fetal health and highlight the necessity of managing oxidative stress during pregnancy to minimize potential risks (Bedell et al., 2021). Future research should further investigate the interactions between oxidative stress, placental function, and fetal development, along with the specific applications of antioxidants in preventing and treating pregnancy complications such as IUGR.

The significant role of oxidative stress in preterm birth and its associated complications reveals how various endogenous and exogenous factors disrupt the balance of the antioxidant system, thereby increasing the risk of preterm delivery. The antioxidant system plays a critical role in fetal development and the newborn’s adaptation to the oxygen-rich extrauterine environment, underscoring the close relationship between preterm birth, immature antioxidant system development, and elevated oxidative stress (Davis and Auten, 2010). Both endogenous and exogenous oxidative stress triggers, such as heavy metal exposure (Ahamed et al., 2009), environmental pollutants, and inflammation (Agarwal et al., 2018), can impair placental function and lead to adverse pregnancy outcomes, including preterm birth (Joo et al., 2021). Research into the specific mechanisms of preterm birth has highlighted the importance of mitochondrial oxidative stress in both preterm delivery and fetal brain injury, while exploring potential therapies aimed at mitigating oxidative stress and inflammation through the induction of the Nrf2 signaling pathway (Chen et al., 2024). Additionally, studies have examined the impact of fetal sex and prenatal glucocorticoid exposure on placental antioxidant balance, indicating that male fetuses may face a higher oxidative stress risk following glucocorticoid exposure (Stark et al., 2011). Multiple studies have established a strong link between preterm birth and oxidative stress by detecting oxidative stress markers in placental tissue and umbilical cord blood. Changes in lipid peroxidation products and antioxidant enzyme activity in preterm placentas (Zadrozna et al., 2009; Ferguson et al., 2015), along with elevated levels of oxidative stress markers like isoprostane in umbilical cord blood (Perrone et al., 2016), suggest a connection to preterm birth. The potential role of antioxidants in preventing preterm birth has been investigated, although certain antioxidants, such as ω-3 polyunsaturated fatty acids, may exhibit pro-oxidant effects under specific conditions, highlighting the need for careful consideration of timing and dosage (Stark et al., 2013; Boulis et al., 2014). On the other hand, some compounds with potential antioxidant and anti-inflammatory properties, such as hydroxylated fullerenes and nicotinamide, have shown promise in preventing preterm birth in animal models (Wakimoto et al., 2015; Lappas and Permezel, 2011). Preterm newborns face unique challenges related to iron metabolism and oxidative stress, given their distinct iron handling and insufficient antioxidant capacity. These newborns are at risk of both iron deficiency and iron overload, necessitating individualized management strategies that take into account multiple factors (Raffaeli et al., 2020). Comprehensive exploration of oxidative stress mechanisms in preterm birth and its related complications offers new insights into the pathophysiology of preterm birth, while providing valuable guidance for future prevention and treatment strategies. Further research should focus on the development of the antioxidant system, the mechanisms of oxidative stress triggers, and the application of antioxidants in preterm birth prevention, with the aim of creating more effective prevention and treatment approaches.

The clustering of terms like “DNA-Damage,” “Insulin-Resistance,” and “Obesity” pointed to strong connections between oxidative stress and broader pathological processes. These connections extended from molecular mechanisms, such as DNA damage (Fujimaki et al., 2011; Singh et al., 2020), to metabolic disruptions like insulin resistance (Feng et al., 2020; Scioscia et al., 2009), further encompassing the public health challenge of obesity. The analysis underscored the complexity and significance of oxidative stress in pregnancy and related diseases, demonstrating its far-reaching impact. Keywords related to maternal health, such as “Pregnant-Women”,“Gestational Diabetes Mellitus” and “Maternal Obesity” prominently appeared in the green cluster, highlighting the impact of oxidative stress not only on fetal health but also on maternal wellbeing. Oxidative stress was recognized as a teratogenic mechanism during embryonic development, particularly in diabetic embryopathy (Coughlan et al., 2004). Studies identified significant alterations in oxidative stress in the placentas of patients with GDM, with elevated levels of nitrotyrosine serving as direct evidence of oxidative stress (Lyall et al., 1998). Additionally, oxidative stress negatively influenced the expression of inflammatory cytokines and antioxidant enzymes in the placentas of GDM patients, though these effects were less evident in adipose tissue (Lappas et al., 2010). To explore potential treatments, various natural extracts, such as mulberry, acacia, and ginkgo leaf extracts, were evaluated for their effects on maternal and fetal outcomes, oxidative stress levels, and lipid profiles in GDM rat models (Volpato et al., 2011; Volpato et al., 2008; Rudge et al., 2007). The findings suggested the potential of these natural extracts to alleviate oxidative stress and improve pregnancy outcomes. Moreover, oxidative stress was found to enhance the activity of matrix metalloproteinases-2 and -9 in the placenta-fetal unit of diabetic rats, potentially exacerbating placental dysfunction (Pustovrh et al., 2005). Beyond natural extracts, research investigated the influence of both endogenous and exogenous oxidative stress triggers on adverse pregnancy outcomes, including preeclampsia, fetal growth restriction, GDM, and preterm birth (Joo et al., 2021). Studies also examined the relationship between adiponectin and oxidative stress markers in GDM patients and their newborns (Shang et al., 2018), as well as oxidative and antioxidant status in GDM patients diagnosed under International Association of the Diabetes and Pregnancy Study Groups criteria (Shang et al., 2015). A cohort study conducted in the Thai population further compared oxidative stress biomarkers between GDM and non-GDM patients, finding a significant association between GDM and inflammatory processes, reflected in higher oxidative stress and apoptosis markers (Rueangdetnarong et al., 2018).

To identify new therapeutic targets, interactions among oxidative stress, endoplasmic reticulum stress, inflammation, mitochondrial function, and signaling pathways were explored. Apocynin mitigated oxidative stress and inflammation in GDM by inhibiting the TLR4/NF-κB signaling pathway (Liu et al., 2020). Cryptotanshinone significantly reduced blood glucose levels, oxidative stress, inflammation, and NF-κB activation in GDM mice, while increasing insulin levels in the placenta and blood (Wang N. et al., 2020). Asperulosidic acid alleviated oxidative stress and inflammation in the GDM placenta by suppressing NF-κB and MAPK signaling pathways (Wu et al., 2022). Additionally, studies assessed the potential benefits of physical activity (Cid and Gonzalez, 2016), specific nutrients like copper (Ergaz et al., 2014)and lutein (Lorenzoni et al., 2013), and the drug nigericin in reducing oxidative stress in GDM patients. Notably, increased placental and fetal lipoprotein-associated phospholipase A2 in GDM patients might offer protection against oxidative stress (Schliefsteiner et al., 2017), The upregulation of nuclear factor erythroid 2-related factor 2 and antioxidant enzymes in the GDM placenta could represent protective mechanisms against oxidative stress (Manoharan et al., 2019). However, not all studies supported the efficacy of these protective mechanisms. For instance, while vitamins and antioxidants reduced fetal oxidative stress, they failed to restore normal growth (Ornoy et al., 2009).

Research also examined the impact of GDM on neonatal cardiovascular health, particularly the increased intima-media thickness of the aorta and the role of oxidative stress in this process (Triantafyllidou et al., 2023). The roles of oxidative stress, fatty acids, and neurotrophic factors in GDM were explored (Jadhav et al., 2020), along with the association between GDM, autism spectrum traits, and attention deficit hyperactivity disorder symptoms, although placental inflammation and oxidative stress cytokines were not found to mediate these associations (Zhu et al., 2021). In summary, this series of studies provides rich data support for understanding the role of oxidative stress in GDM and its related complications, and offers potential targets for the development of new therapeutic strategies.

GDM (Gauster et al., 2017; Lappas et al., 2011) and obesity (Duan et al., 2018; Hu et al., 2021) both significantly increase oxidative stress in the placenta, posing risks to maternal and fetal health during pregnancy. Research indicates that maternal obesity is not only associated with metabolic abnormalities and reduced antioxidant capacity in pregnant women but may also affect placental function, leading to fetal oxidative stress and metabolic alterations (Malti et al., 2014; Zavalza-Gomez, 2011; Ballesteros-Guzman et al., 2019). Elevated levels of oxidative stress markers, such as malondialdehyde, protein carbonyls, nitric oxide, and superoxide anions, have been observed in the placentas of obese pregnant women, alongside decreased levels of antioxidants like reduced glutathione and superoxide dismutase. This imbalance in redox status could have long-term effects on fetal metabolic and immune programming (Malti et al., 2014; Hernandez-Trejo et al., 2017). Additionally, obesity may contribute to placental mitochondrial dysfunction and impaired angiogenesis, further exacerbating oxidative stress in the placenta (Hu C. et al., 2019; Napso et al., 2022). Notably, alterations in the gut microbiota of obese pregnant women have been linked to increased oxidative stress in the placenta, suggesting a potential role for gut microbiota in obesity-mediated placental dysfunction (Hu et al., 2021). To explore therapeutic interventions, studies have evaluated the effects of resveratrol supplementation on oxidative stress in obese pregnant women and their fetuses. The results demonstrated that resveratrol improved maternal metabolic status and significantly reduced oxidative stress in the placenta and liver, offering new strategies for managing obesity during pregnancy (Rodriguez-Gonzalez et al., 2022). Research has also focused on the impact of maternal obesity on the renal health of offspring. Offspring of obese pregnant women may experience lipid accumulation, inflammation, oxidative stress, and fibrosis in the kidneys, increasing the risk of developing kidney diseases (Phengpol et al., 2023). An exploratory study evaluated the effects of physical activity and sedentary time during pregnancy on placental oxidative stress markers in obese women. The findings suggest that increased physical activity and reduced sedentary time may correlate with lower expression levels of certain oxidative stress markers in the placenta, underscoring the importance of lifestyle interventions during pregnancy (Zafaranieh et al., 2022). Obesity significantly influences oxidative stress in both pregnant women and their fetuses, potentially involving complex physiological and pathological mechanisms. Future research should further investigate the role of obesity-mediated oxidative stress in pregnancy complications and develop effective interventions to improve health outcomes for obese pregnant women and their offspring. These insights highlighted the need for comprehensive strategies to assess and manage oxidative stress during pregnancy (Bedell et al., 2021), aiming for an integrated approach that protects both maternal and fetal health.

The blue cluster primarily focuses on pregnancy-related diseases and their underlying pathophysiological mechanisms. The cluster, with a central focus on “Preeclampsia,” strongly associated with keywords like “Hypertension,” “Nitric-Oxide,” and “Nitric-Oxide Synthase,” highlighting their frequent co-mention in the literature. This co-occurrence indicated the critical roles and interactions of these factors in preeclampsia’s pathophysiology. Research linked preeclampsia to significant oxidative stress, which reduced nitric oxide (NO) bioavailability (Nunes et al., 2021) through the inhibition of endothelial nitric oxide synthase (eNOS) activity (Amaral et al., 2013). Vitamin D deficiency further aggravated the reduction of NO by influencing oxidative stress responses in human umbilical vein endothelial cells (Nunes et al., 2021). L-arginine depletion in preeclampsia contributed to eNOS conversion into an oxidant form, decreasing NO production, while oxidative products such as 4-oxo-2(E)-nonenal exacerbated eNOS dysfunction (Guerby et al., 2019). Inhibition of inducible nitric oxide synthase demonstrated potential for blood pressure reduction in experimental studies (Amaral et al., 2013), and antioxidants like N-acetylcysteine helped restore NO-mediated function in the placenta of preeclampsia patients (Bisseling et al., 2004). Heat shock protein 70 (Barut et al., 2010), homocysteine, and asymmetric dimethylarginine also played key roles in regulating NO metabolism and the development of preeclampsia (Demir et al., 2012). Overall, the imbalance between NO and reactive oxygen species, particularly oxidative stress-induced eNOS inhibition, emerged as a critical aspect of preeclampsia’s pathogenesis. Future studies should further investigate the molecular mechanisms and potential interventions related to this imbalance.

The frequent appearance of keywords such as “Pathogenesis,” “Endothelial Growth-Factor,” “Blood-Flow,” “Necrosis-Factor-Alpha,” “Blood-Pressure,” and “Endothelial Dysfunction” underscored the central role of vascular endothelial dysfunction (Sanchez-Aranguren et al., 2014; Cindrova-Davies, 2009), inflammatory responses (Tenorio et al., 2019; Harmon et al., 2016; Michalczyk et al., 2020), and blood flow regulation disorders (Parra et al., 2005; Yoshihara et al., 2022) in preeclampsia’s pathogenesis and related hypertensive diseases. These interactions collectively drove the progression of preeclampsia. Keywords like “Placental Oxidative Stress,” “Cytokines,” “Fetal Growth Restriction,” “Trophoblast Invasion,” and “Angiogenic Factors” revealed critical aspects of preeclampsia’s complex pathophysiology, including placental dysfunction (Biron-Shental et al., 2010), oxidative stress responses (Marin et al., 2020), abnormal angiogenesis (Turpin et al., 2015), and fetal growth restriction (Takagi et al., 2004). The role of the placenta in preeclampsia pathology and the impact of oxidative stress (Anto et al., 2023), cytokine network imbalances (Rusterholz et al., 2007; Stark, 1993), trophoblast cell dysfunction (Wang GJ. et al., 2020), and angiogenesis disorders (Anto et al., 2024; Edvinsson et al., 2022) were emphasized.

Finally, the appearance of keywords such as “Tumor-Necrosis-Factor,” “Tyrosine Kinase-1,” and “Endoplasmic-Reticulum Stress” suggested potential roles for immune-inflammatory responses (Kim et al., 2017), signaling pathway abnormalities (Kim et al., 2017; Burke et al., 2016), and cellular stress responses (Redman et al., 2022) in preeclampsia’s pathogenesis, further emphasizing the complexity of this multifactorial disease involving widespread biological processes and molecular network imbalances (Burton et al., 2019).

The keywords in the red cluster form a highly integrated knowledge network closely related to cell biology, oxidative stress, and mechanisms of related diseases. This cluster centers around high-frequency keywords such as ‘Expression,’ ‘Apoptosis,’ ‘Cells,’ and ‘Trophoblast,’ highlighting the central roles of cellular expression regulation, cell apoptosis, and trophoblast cells in these research domains. These keywords not only represent fundamental processes in cell biology but also clearly indicate the critical roles of these processes within complex physiological and pathological environments, particularly in the contexts of pregnancy and placental function.

Additionally, the appearance of keywords such as “*In-Vitro*,” “Hypoxia,” “Activation,” “Growth,” and “Mitochondria” further underscores the significant impacts of *in vitro* experimental models (Depoix et al., 2017), hypoxic environments (Zhao et al., 2021) on cellular functions, and the crucial roles of mitochondria in cell growth (Ma et al., 2019), energy metabolism (Napso et al., 2022), and stress responses (Burton et al., 2017). The co-occurrence of these keywords reveals multiple important aspects in cell biology research, including the establishment of experimental models, cellular adaptive responses to environmental conditions, and the pivotal role of organelles, especially mitochondria, in maintaining cellular homeostasis.

Notably, the co-occurrence of keywords such as “Reactive Oxygen Species,” “Stress,” “Disease,” “Angiogenesis,” and “Oxygen” reveals the close relationship between oxidative stress responses, abnormal angiogenesis, and the placenta. Research has shown that ROS not only participates in physiological processes of normal pregnancy, such as placental angiogenesis (Yang et al., 2022) and trophoblast function (Walker et al., 2020), but also plays a key role in various pregnancy complications, including preeclampsia [(Matsubara et al., 2015), (Matsubara et al., 2010)], intrauterine growth restriction (Luo et al., 2018), and fetal loss (Zhang et al., 2019). ROS mediates oxidative stress responses by regulating signaling pathways (Adebambo et al., 2018), affecting gene expression (Wang B. et al., 2020), and impacting cellular functions (Adebambo et al., 2018), thereby influencing pregnancy outcomes. Furthermore, insulin resistance and elevated androgen levels (Hu M. et al., 2019) can increase ROS production, further exacerbating the risk of pregnancy complications. Notably, the application of antioxidants has shown potential in alleviating ROS-mediated damage (Al-Gubory et al., 2010; Khera et al., 2017), providing new insights for clinical intervention. Future research should continue to explore the specific mechanisms of ROS in pregnancy and develop more effective antioxidant therapies to improve pregnancy outcomes and safeguard maternal and fetal health.

The balance between pro-angiogenic and anti-angiogenic factors is crucial for maintaining normal pregnancy. In particular, pathological states such as preeclampsia and intrauterine growth restriction are associated with significant inhibition of angiogenesis (Shi et al., 2021), along with increased oxidative stress (Anto et al., 2024) and changes in the expression of angiogenesis-related genes (Fortis et al., 2018). Additionally, factors such as maternal nutritional status (Anto et al., 2023), environmental factors (Bai et al., 2022), pharmacological interventions (Soobryan et al., 2017), and genetic variations (Fortis et al., 2018) all impact placental angiogenesis. Notably, certain natural compounds (Park et al., 2020)and drugs (Doganlar et al., 2019) have shown potential in promoting angiogenesis, mitigating oxidative stress, and improving pregnancy outcomes. Future research should further explore the specific mechanisms of angiogenesis in pregnancy and develop more effective intervention strategies to improve the prognosis of patients with pregnancy complications by modulating the angiogenesis process.

Furthermore, the clustering of keywords such as “Toxicity,” “Liver,” “Damage,” and “Dysfunction” reveals the broad pathological processes potentially involved in oxidative stress and cellular dysfunction. These processes include the toxic effects and mechanisms of various chemicals (Abdollahzade et al., 2021; Cattani et al., 2014), environmental pollutants (Saben et al., 2014), pharmaceuticals (Lu et al., 2023), and nanomaterials (Wang and Wang, 2020) on the placenta, trophoblast cells, embryo, and fetus. They collectively highlight the central roles of oxidative stress (Huang et al., 2018), apoptosis (Ali et al., 2021), genotoxicity (Kostic et al., 2022), endocrine disruption (Perez-Albaladejo et al., 2017), and mitochondrial dysfunction (Naav et al., 2020) in these toxic effects. Notably, research emphasizes the placenta as a critical target for these toxic effects and its key role in protecting the fetus from external harmful substances. Simultaneously, these studies reveal the potential applications of various antioxidants (Jan et al., 2011), nutrients (Hassoun et al., 1997), and other bioactive substances (Lee et al., 2023) in mitigating or counteracting these toxic effects. The keyword “Liver” primarily focuses on liver diseases (Pillai and Gupta, 2005), tumors (Puatanachokchai et al., 2006), and their prevention, progression, and treatment (Phannasorn et al., 2022). The terms “Damage” and “Dysfunction” indicate that the impact of oxidative stress is not confined to a specific disease or organ but widely affects multiple systems and organ functions. This co-occurrence of keywords not only expands our understanding of the spectrum of oxidative stress-related diseases but also emphasizes the core role of cellular dysfunction in these diseases, suggesting that future research should focus more on the mechanisms of oxidative stress-induced cellular dysfunction and its specific roles in various diseases.

The co-occurrence of keywords such as “Protein,” “Differentiation,” “Proliferation,” “Inhibition,” “Invasion,” “Receptor,” and “Autophagy” further enriches our understanding of cellular biological processes. These keywords address mechanisms across several layers, including protein functions (Colleoni et al., 2013), cell differentiation and proliferation (Bolnick et al., 2017), signal transduction (Rundlof and Arner, 2004), and cell invasion and migration (Yang and Shang, 2021). The close association of these keywords reveals multiple complex aspects of cell biology research, including the critical roles of proteins in determining cell fate (Rundlof and Arner, 2004), regulatory mechanisms of cell proliferation and differentiation (Dai and Lu, 2022), and the complexity of intercellular interactions and signal transduction (Murphy et al., 2008). Additionally, the appearance of keywords such as “Pregnancies,” “Messenger-RNA,” “Identification,” “ROS,” and “Gene” highlights the significant roles of oxidative stress, gene expression regulation, and reactive oxygen species in cell fate decisions during pregnancy. These keywords reveal the complexity and uniqueness of cellular biological processes under the special physiological state of pregnancy and the critical roles of oxidative stress and gene expression regulation in this process.

The yellow cluster focuses on oxidative stress, antioxidant mechanisms, and related biomarkers, centering around high-frequency keywords like “Lipid-Peroxidation” and “Antioxidant.” This cluster primarily explores the formation and effects of antioxidants and lipid peroxidation in human placental and fetal tissues. Specifically, it covers studies on free radical scavenging enzyme activity and lipid peroxidation in placental tissues of miscarriage patients (Biri et al., 2006), NADPH and iron-dependent lipid peroxidation in human placental microsomes, and lipid peroxidation, antioxidant defenses, and acid-base status in umbilical cord blood at birth (Milczarek et al., 2007). Additionally, the cluster investigates the effects of various substances, such as melatonin (Milczarek et al., 2000), pollutants (Lee et al., 2015), and drugs (Rojas et al., 2022) on lipid peroxidation in the placenta and fetus, as well as changes in lipid peroxidation and antioxidant enzyme activity under different physiological and pathological conditions (Johnston et al., 2016; Loverro et al., 1996). Collectively, these studies reveal the complexity of lipid peroxidation and antioxidant states in placental and fetal tissues and their crucial roles in pregnancy and fetal development.

The emergence of keywords such as “Human Placenta” and “Women” indicates that this research network may particularly focus on oxidative stress and antioxidant mechanisms within the context of female physiology, especially within the placenta. The co-occurrence of keywords like “Plasma,” “Superoxide-Dismutase,” “Vitamin-E,” “Glutathione,” and “Selenium” further underscores the critical role of antioxidant defense systems in maintaining cellular homeostasis (Wang and Walsh, 2001; Liu et al., 2010) and highlights the potential biomarker value of antioxidants and enzymes present in plasma (Rani et al., 2010). The appearance of the term “Supplementation” suggests the application of antioxidants as an intervention strategy in related research (Poston et al., 2011; Pressman et al., 2003). Additionally, the clustering of keywords such as “Melatonin,” “Free-Radicals,” and “Antioxidant Enzymes” reveals the production of free radicals during oxidative stress (Lista et al., 2010), the occurrence of lipid peroxidation (Gupta et al., 2005), and the pivotal role of antioxidant enzymes in scavenging free radicals (Vanderlelie et al., 2005) and protecting cells from oxidative damage (Okatani et al., 2001). Notably, the appearance of “Messenger-RNA Expression” and “Alpha-Tocopherol” adds a new dimension to this research network, indicating a potential connection between oxidative stress and gene expression regulation (Than et al., 2009), as well as the specific mechanisms through which particular antioxidants contribute to cellular protection (Vieira et al., 2018). Finally, although keywords like “Thromboxane” and “Acid” appear less frequently in this cluster, they represent broader areas of oxidative stress research, such as the relationship between oxidative stress and thrombosis (Vaughan and Walsh, 2005), and acid-base balance in physiological and pathological processes (Kinalski et al., 2001).

Several core themes have emerged as significant research hotspots, providing clear directions for researchers. Oxidative stress and lipid peroxidation processes have been longstanding focal points. The close relationship between “lipid peroxidation” and “oxidative stress” highlights the potential harm lipid peroxidation products inflict on placental function during oxidative stress, offering critical insights for the development of subsequent intervention strategies. Additionally, the role of antioxidants, particularly natural or synthetic agents such as “vitamin E,” has been widely recognized for mitigating oxidative stress and protecting placental health, providing new perspectives on nutritional supplementation and therapeutic approaches during pregnancy. Pregnancy-related disorders have become key research priorities due to their strong association with oxidative stress. Understanding these conditions is essential for elucidating disease mechanisms and optimizing clinical management. The complex roles of gene expression and regulation in placental oxidative stress responses have also gained clarity. The frequent appearance of “gene expression” and related keywords underscores the importance of genetic research in revealing molecular mechanisms underlying oxidative stress, prompting researchers to focus more on genetic factors in this field.

Emerging directions and fields are also worth attention. Pathophysiology and mechanism exploration are gaining prominence, with the rise of keywords such as “pathophysiology” and “mechanism” indicating that deeper investigation into the pathophysiological mechanisms of placental oxidative stress could provide theoretical support for precision therapies. Emerging risk factors and environmental influences have garnered increasing attention, with the appearance of keywords like “obesity,” “bisphenol A,” and “air pollution” revealing potential threats posed by environmental factors to placental health and pregnancy outcomes, thus offering a scientific basis for public health policies. The growth of cellular biology and stem cell research, especially the increased focus on “cell,” “stem cell,” and “autophagy,” highlights the growing interest in cellular processes related to placental oxidative stress. Stem cell therapy and autophagy regulation could open new avenues for future therapeutic strategies. Furthermore, inflammation and immune regulation remain enduring research frontiers, with the complex interactions between placental oxidative stress and these processes being increasingly revealed. The sustained high frequency and recent rise of keywords such as “inflammatory cytokine” and “inflammation” underscore the critical role inflammation and immune regulation play in maintaining placental homeostasis and preventing related diseases, encouraging further exploration in this area.

Based on the analysis of core themes and research frontiers, this paper provides a scientific basis for policymakers to develop public health policies. Emphasis should be placed on the role of oxidative stress and lipid peroxidation in adverse pregnancy outcomes, encouraging further research to design effective interventions. Additionally, addressing emerging risk factors such as obesity, bisphenol A, and air pollution is crucial. Policymakers should implement targeted public health measures to alleviate their detrimental effects on placental health and pregnancy outcomes. Potential actions include advocating for stricter air pollution regulations and launching public health education campaigns to increase awareness of the risks associated with obesity and environmental pollutants.

Employing bibliometrics to explore the field of “placental oxidative stress” offers a novel perspective and facilitates a deeper understanding of its trends. Unlike traditional literature reviews, the study innovatively employed multiple bibliometric tools such as CiteSpace, VOSviewer, and the R package bibliometrix. The integrated use of these tools enabled a comprehensive and systematic extraction and analysis of data, revealing key research dynamics in the field of placental oxidative stress. By combining systematic searches with quantitative statistical analysis, the approach provided a more data-driven and quantitative view of the research landscape.

However, the study had certain limitations. First, the data were sourced solely from the Web of Science Core Collection, potentially overlooking relevant information from other databases such as Embase and MEDLINE. This choice stemmed from the fact that the Web of Science Core Collection is the most commonly used database for bibliometric analysis, offering timely and comprehensive citation updates. Nonetheless, challenges in integrating data from different sources persist due to limitations in the analytical software. Second, the sensitivity of the analysis algorithms may have missed emerging topics related to “placental oxidative stress.” Despite employing multiple software packages for analysis, the information provided remained constrained by the limitations of bibliometric algorithms. Future advancements in bibliometric methods may help address these challenges.

Additional challenges arose during the bibliometric analysis. First, scientometric research primarily relies on co-citation analysis, which can be influenced by “citation distortion”—the selective use of citations to support unverified scientific claims. This issue, although detectable through close examination of research hotspots, remains a notable limitation. Second, limiting the search to the Web of Science Core Collection may have restricted the range of publication types retrieved. In many databases such as PubMed and Embase, full-text references and citation lists are unavailable, complicating efforts to merge references from different databases and often requiring significant manual intervention. Future software developments may enable the simultaneous analysis of results from various databases while reliably removing duplicates automatically. Third, the analysis did not cover all publications related to “placental oxidative stress,” as only original articles and reviews were included, and the Web of Science’s stricter inclusion criteria for scientific journals, compared to databases like PubMed, further constrained the scope. Lastly, the inherent lag in publications and subsequent citations made it difficult to capture the latest research trends. However, the impact of this lag was significantly reduced by considering the context in which articles were cited and who conducted the citations, as cited articles might have been recently published.

In summary, despite its limitations, the bibliometric analysis employed in this study provided valuable insights into research trends in the field of placental oxidative stress and suggested new directions for future research. To further improve the analysis in this area, future studies should consider integrating multiple databases and analytical methods for more comprehensive and in-depth insights.

## 5 Conclusion

This study provides an in-depth bibliometric analysis of the field of “placental oxidative stress” aiming to reveal trends, research hotspots, and future directions. The study identifies key research hotspots focused on oxidative stress and lipid peroxidation processes, with “lipid peroxidation” as a critical event indicating potential risks to placental function. The role of antioxidants, particularly natural or synthetic substances such as vitamin E, in mitigating oxidative stress and protecting placental health is well-recognized, offering new insights for nutritional supplementation and therapeutic strategies during pregnancy. Pregnancy-related diseases, particularly “preeclampsia” and “pregnancy-induced hypertension,” are significant research focuses due to their close association with oxidative stress, which is crucial for understanding disease mechanisms and optimizing clinical management. Additionally, the complex role of gene expression and regulation in the placental oxidative stress response is increasingly evident, highlighting the importance of genetic-level research in elucidating molecular mechanisms. Emerging research directions include exploring pathophysiological mechanisms, focusing on new risk factors and environmental impacts, advancing cell biology and stem cell research, and continuing to investigate inflammation and immune regulation. These developments are expected to enhance our understanding of placental oxidative stress and advance therapeutic strategies.

## Data Availability

The original contributions presented in the study are included in the article/supplementary material, further inquiries can be directed to the corresponding authors.
